# The Role of Candidate Polymorphisms in Drug Transporter Genes on High‐Dose Methotrexate in the Consolidation Phase of the AIEOP‐BFM ALL 2009 Protocol

**DOI:** 10.1111/cts.70136

**Published:** 2025-01-31

**Authors:** Stefania Braidotti, Giulia Zudeh, Raffaella Franca, Valentina Kiren, Antonella Colombini, Laura Rachele Bettini, Erica Brivio, Franco Locatelli, Luciana Vinti, Nicoletta Bertorello, Franca Fagioli, Daniela Silvestri, Maria Grazia Valsecchi, Giuliana Decorti, Gabriele Stocco, Marco Rabusin

**Affiliations:** ^1^ Department of Pediatrics Institute for Maternal and Child Health ‐ IRCCS Burlo Garofolo Trieste Italy; ^2^ Department of Translational and Advanced Diagnostics Institute for Maternal and Child Health ‐ IRCCS Burlo Garofolo Trieste Italy; ^3^ Department of Medical, Surgical and Health Sciences University of Trieste Trieste Italy; ^4^ Department of Pediatrics, IRCCS San Gerardo dei Tintori Monza Italy; ^5^ University of Milano‐Bicocca Milan Italy; ^6^ Department of Pediatric Oncology Erasmus MC‐Sophia Children's Hospital Rotterdam Rotterdam The Netherlands; ^7^ Princess Máxima Center for Pediatric Oncology Utrecht The Netherlands; ^8^ Department of Hematology, Oncology and of Cell and Gene Therapy IRCCS Ospedale Pediatrico Bambino Gesú, Catholic University of the Sacred Heart Rome Italy; ^9^ Paediatric Onco‐Haematology Department Regina Margherita Children's Hospital Turin Italy; ^10^ Department of Public Health and Pediatrics University of Turin Turin Italy; ^11^ Bicocca Centre of Bioinformatics, Biostatistics and Bioimaging, School of Medicine and Surgery University of Milano Bicocca Milano Italy

**Keywords:** drug transporters, high‐dose methotrexate pharmacokinetics, pediatric acute lymphoblastic leukemia, pharmacogenetics

## Abstract

High‐dose methotrexate (HD‐MTX) infusions are commonly used to consolidate remission in children with acute lymphoblastic leukemia (ALL). We investigate the potential role of candidate polymorphisms in *SLCO1B1* (rs4149056 and rs2306283), *ABCB1* (rs1045642), *ABCC2* (rs717620), *ABCC3* (rs9895420), and *ABCC4* (rs7317112) drug transporters genes on HD‐MTX pharmacokinetics and patients' outcome (meant both as relapse and drug‐related toxicities) in an Italian cohort of 204 ALL pediatric patients treated according to the AIEOP‐BFM ALL 2009 protocol. TaqMan SNP genotyping assays determined patient's genotypes. Measurements of HD‐MTX plasma concentration were available for 814 HD‐MTX courses in 204 patients; MTX clearance was estimated by a two‐compartmental linear pharmacokinetic model with first‐order elimination and a Bayesian approach, via ADAPT. Independent contributions of age and *ABCC4* SNP rs7317112 (A>G, intronic) on MTX clearance were detected in a multivariate analysis (*p* = 1.57 × 10^−8^ and *p* = 2.06 × 10^−5^, respectively), suggesting a delayed elimination of the drug in older patients and an accelerated one in carriers of the variant GG genotype. After multiple corrections, the association between *ABCC2* SNP rs717620 (−24 C>T) and severe hematological toxicity was found (*p* < 0.005). Moreover, *SLCO1B1* SNP rs4149056 (c.521T>C, p.V174A) affected patients' outcomes: carriers of the variant C allele presented a reduced risk of relapse compared to wild‐type TT (hazard risk: 0.27, 95% confidence interval [CI]: 0.08–0.90, *p* = 0.037). Taken together, these data highlighted the importance of variants in drug transporters genes on HD‐MTX disposition in the AIEOP‐BFM ALL 2009 protocol consolidation phase, and their putative role as predictive markers of outcome.

**Summary**

- What is the current knowledge on the topic?
  ○ High‐dose methotrexate (HD‐MTX) is widely used to consolidate remission in pediatric acute lymphoblastic leukemia (ALL). Single nucleotide polymorphisms (SNPs) in drug transporter genes may affect the pharmacokinetics and toxicity of HD‐MTX and thus the clinical outcome.
- What question did this study address?
  ○ This study investigated the role of candidate SNPs in drug transporter genes (i.e., *SLCO1B1* rs4149056 and rs2306283, *ABCB1* rs1045642, *ABCC2* rs717620, *ABCC3* rs9895420, and *ABCC4* rs7317112) and/or of HD‐MTX pharmacokinetics on patients' outcome for the first time in an Italian cohort of ALL pediatric patients treated according to the AIEOP‐BFM ALL 2009 protocol.
- What does this study add to our knowledge?
  ○ This study provided evidence that genetic variants in drug transporter genes significantly affect HD‐MTX pharmacokinetics and patient outcomes, highlighting their putative role as predictive markers of outcome in the AIEOP‐BFM ALL 2009 protocol consolidation phase:
    - *ABCC4* SNP (rs7317112): Carriers of the variant GG genotype showed accelerated MTX clearance;
    - *ABCC2* SNP (rs717620): Carriers of the T‐variant allele showed an increased risk of drug‐related adverse effects, in particular of hematological toxicities;
    - *SLCO1B1* SNP (rs4149056): Carriers of the C‐variant allele showed lower risk of relapse.
- How might this change clinical pharmacology or translational science?
  ○ Our findings emphasize the importance of genetic evaluation for drug transporter polymorphisms in pediatric ALL patients undergoing HD‐MTX treatment in the therapeutic context of the AIEOP‐BFM consolidation phase. Identifying patients with significant genetic variants can help personalize treatments, potentially improving therapeutic outcomes and reducing drug‐related toxicities. This approach can lead to more precise dosing strategies based on individual genetic profiles, advancing the field of pharmacogenomics in pediatric oncohematology and improving patient care.

## 1 Introduction

Acute lymphoblastic leukemia (ALL) is one of the most frequent pediatric malignant conditions and represents approximately 80% of leukemia cases occurring in childhood [1]. ALL is treated according to long‐lasting (~2 years) polychemotherapeutic protocols, organized in different phases to induce, consolidate, and maintain remission. The most intense treatment is given within the first 4–6 months after diagnosis to eradicate leukemic cells, whereas a prolonged mild myelosuppression is required afterward to achieve a sustained event‐free survival [2].

**FIGURE 1:**
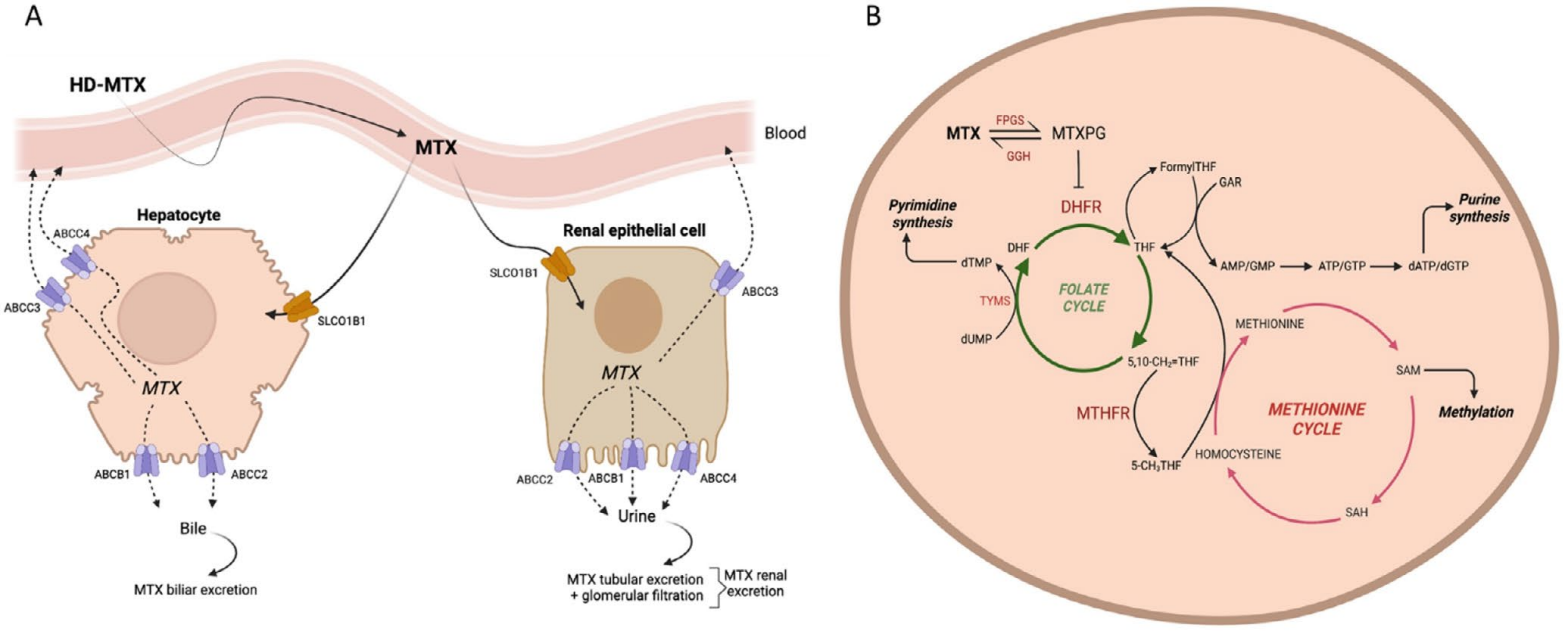
MTX pharmacological pathways shown in a simplified version. (A) Pharmacokinetic pathways; (B) pharmacodynamic pathways. Once in the cells, the folylpolyglutamate synthetase (FPGS) converts MTX into polyglutamates (MTXPGs) that are retained into cells for a prolonged period. MTXPGs are irreversible competitive inhibitors of the target enzyme dihydrofolate reductase (DHFR) that catalyzes the conversion of dihydrofolate (DHF) into tetrahydrofolate (THF), an important cofactor of de novo purine synthesis and a major one‐carbon carrier in the body. Additionally, MTXPGs impair several other enzymes, such as 5,10‐methylenetetrahydrofolate reductase (MTHFR), involved in folate metabolism, DNA replication, and methylation of both DNA and protein and thymidylate synthetase (TYMS), involved in the conversion of deoxyuridine monophosphate (dUMP) to deoxythymidine monophosphate (dTMP). Adapted from pharmGKB (https://www.pharmgkb.org/pathway/PA165816349).

Methotrexate (MTX) is a cornerstone drug in ALL therapy, widely used across all therapeutic phases. During induction, the intrathecal administration of MTX is common for central nervous system treatment and prophylaxis, whereas its systemic exposure is recommended once remission is achieved. In particular, high‐doses MTX (HD‐MTX, i.e., dose greater than 500 mg/m^2^) are intravenously administered to target remaining leukemic blasts in consolidation, whereas low doses of MTX (e.g., 20 mg/m^2^) are weekly administered orally in maintenance to keep patients in immunosuppression [2, 3]. Being hydrophilic, MTX crosses the biological barriers poorly and through active transport systems. It enters cells by influx folate carriers, such as the well‐characterized SLC19A1 in enterocytes and SLCO1B1 in hepatocytes; ABC efflux transporters mediate cellular elimination [4, 5]. In hepatocytes, most of MTX reenters the blood circulation by ABCC3 (also known as multidrug resistance‐associated protein, MRP3) and ABCC4 (MRP4), and only a small portion is excreted into the bile ducts via the apical/canalicular ABCC2 (MRP2) and ABCB1 (also known as P‐glycoprotein or multidrug resistance protein 1, MDR1) [6]. ABCB1 is a ubiquitously expressed pump, and also mediates the active secretion of MTX into urinary filtrate in the proximal tubular cells. ABCC2 and ABCC4 are also located in the apical membrane of the renal epithelial cells, contributing, in this way, to lower body drug exposure; in contrast, ABCC3 is basolateral located in the proximal tubule (Figure 1A) [7, 8]. Once in the cells, MTX is converted into polyglutamates (MTXPGs) that are retained in cells for a prolonged period and act as antimetabolites interfering with the folate pathway in the dividing cells (Figure 1B). Overall, MTXPGs induce the depletion of reduced folates and compromise de novo purine and thymidine synthesis, which are paramount for the survival of leukemic stem cells. These antiproliferative effects are mainly observed for HD‐MTX; the immunosuppressant effects achieved at low doses MTX are less characterized, likely involving other cellular mechanisms than the folate cycle imbalance [9].

Being a nonselective drug, MTX may affect both tumoral and healthy rapidly dividing cells, leading to severe toxicities of normal tissues, and thus to therapy interruption or discontinuation [10, 11, 12]. Incidence and severity of drug‐related adverse reactions depend on exposure intensity [13]. Toxicity of low doses MTX primarily manifests as moderate myelosuppression and hepatotoxicity in ALL patients. In contrast, patients receiving HD‐MTX are at risk of severe hematologic, gastrointestinal, and hepatic side effects; indeed, supplemental folinic acid intravenous infusions are given post HD‐MTX as a “rescue” therapy for healthy cells. Moreover, supportive measurements such as urine alkalinization and hydration are recommended since the massive urine excretion of MTX may exceed drug solubility and lead to drug crystallization and nephrotoxicity. Nonetheless, the troubling occurrence of HD‐MTX‐induced toxicities often results from variability in MTX pharmacokinetics (PK), and remains a concern.

In the last decade, genome‐wide association studies (GWAS) and/or candidate gene analysis have investigated the contribution of single nucleotide polymorphism (SNP) on HD‐MTX PK and clinical outcomes [14, 15, 16]. The role of some genetic variants in *SLCO1B1*, *ABCB1*, *ABCC2*, *ABCC3*, and *ABCC4* genes emerged (Table S1); however, their contribution have never been evaluated in ALL protocols of the European AIEOP‐BFM consortium. Therefore, we investigate the potential role of candidate polymorphisms in *SLCO1B1* (rs4149056 and rs2306283), *ABCB1* (rs1045642), *ABCC2* (rs717620), *ABCC3* (rs9895420), and *ABCC4* (rs7317112) on HD‐MTX PK and on patients' outcome (meant both as relapse and drug‐related toxicities during the consolidation phase) in an Italian cohort of ALL pediatric patients treated according to the AIEOP‐BFM ALL 2009 protocol.

## 2 Materials and Methods

### 2.1 Study Population

HD‐MTX pharmacological parameters were measured in a cohort of newly diagnosed Philadelphia–negative ALL pediatric patients (age: 1–18 years). The cohort comprised 204 ALL pediatric patients enrolled between 2012 and 2017 at the Hemato‐oncological Units of the Hospitals IRCCS “Burlo Garofolo” in Trieste (*n* = 33, 16.2%), “Regina Margherita” in Turin (*n* = 30, 14.7%), “IRCCS San Gerardo dei Tintori” in Monza (*n* = 83, 40.7%), and “Bambino Gesù” in Rome (*n* = 58, 28.4%) in Italy. These patients were treated according to the AIEOP‐BFM ALL 2009 protocol (ClinicalTrials.gov: NCT01117441) and fell in the standard‐ and intermediate‐risk class receiving protocol M as consolidation phase. High‐risk patients were excluded from the study because not eligible to consolidation protocol M; Down Syndrome children were excluded because of their differently planned HD‐MTX therapeutic scheme (a dose of 0.5 g/m^2^ in the first MTX course is recommended), due to their poor drug tolerance and higher frequency of drug‐related adverse effects [17].

Protocol M lasted 56 days and consisted of daily oral administration of mercaptopurine (MP, 25 mg/m^2^) and four courses of HD‐MTX (5 g/m^2^, regardless of age), given every other week since day +8. Dose adjustments were allowed in subsequent courses when children experienced episodes of severe MTX toxicity with the standard dose. HD‐MTX was administered as intravenous infusion over 24 h (h), with a 10% loading dose over 0.5 h. By protocol, MTX plasma levels were measured at 24 h (end of infusion) and 42 h; in case of delayed drug excretion (i.e., MTX concentration greater than 0.5 μM), additional monitoring was planned every 6 h. Standard leucovorin rescue (7.5 mg/m^2^) occurred at 42 h and every 6 h, with a minimum of three doses or until MTX plasma concentration was lower than 0.25 μM; leucovorin dosage could be increased if necessary according to AIEOP‐BFM ALL 2009 protocol guidelines. Standard supportive care guidelines included hyperhydration (3000 mL/ m^2^/day) and urine alkalinization (pH > 7), continued until MTX plasma concentration lower than 0.25 μM. Information of other concomitant medications used in consolidation was not available.

The study was approved by the Institutional Review Board (or Ethics Committee) of IRCCS Burlo Garofolo (Protocol number CE/V 135; March 5, 2012) and appropriate informed consent was obtained from patients/donors and/or their parents or guardians.

### 2.2 DNA Extraction and Pharmacogenetics Analysis

Total genomic DNA was isolated from patient peripheral blood collected in remission using a commercial kit (GenElute Blood Genomic DNA Kit, Sigma‐Aldrich, Milan, Italy) according to the manufacturer's instruction, and stored at −20°C until use. TaqMan SNP genotyping assays were used (*SLCO1B1* rs2306283 assay ID: C__1901697_20, *SLCO1B1* rs4149056: C__30633906_10, *ABCB1* rs1045642: C__7586657_20, *ABCC2* rs717620: C__2814642_10, *ABCC3* rs9895420: C__27850763_10, *ABCC4* rs7317112: C__29165801_20; Applied Biosystems, Foster City, CA).

### 2.3 Pharmacokinetics Analysis

MTX plasma concentration were measured by Syva methotrexate assay kit (Siemens). Measurements of MTX plasma concentration were available for 814 HD‐MTX courses in 204 patients. Four patients did not receive infusion at 5 g/m^2^ in their first course for clinical decision, and were excluded from the analysis to avoid confounding clinical situation. MTX clearance was estimated based on at least two MTX plasma concentrations per course up to all available measurements fitted to a two‐compartmental linear pharmacokinetic model with first‐order elimination and a Bayesian approach, via ADAPT software (University of Southern California, Los Angeles, CA). Both dosing intervals (0–0.5 h and 0.5–24 h) with individual dosing rates were included in the model. Model parameters were kindly provided by Prof. Carl Panetta and Prof. William Evans (Saint Jude Children Research Hospital, Memphis, Tennessee, USA). Initial estimates of apparent volume of distribution and rate constants between compartments were set as follows: *V* = 9.03 ± 4.70 L/m^2^, ke = 0.7 ± 0.22 h^−1^, kcp = 0.08 ± 0.050 h^−1^, and kpc = 0.11 ± 0.0038 h^−1^ [18]. Two different bicompartmental models (LR or SHR) have been used to analyze data based on the HD‐MTX dose actually infused (< 4 g/m^2^ or ≥ 4 g/m^2^, respectively).

### 2.4 Clinical Data

Clinical data were retrieved from the AIEOP central database on February 2022, whereas pediatricians collected toxicities retrospectively, blinded to results of pharmacological analysis. At February 2019, all patients were out of therapy. Relapses were defined according to routine clinical practice and occurred at any time in the follow‐up period (Table 1). Hematological toxicity (HEM) and gastrointestinal toxicities (GI) were graded referring to a simplified version of the National Cancer Institute‐Common Terminology Criteria scales, regardless of their time of occurrence during consolidation. Severe HEM toxicities (grade ≥ 3) consisted in bone marrow suppression, with episodes of hemoglobin < 8 g/dL or white blood cells (WBC) < 2 × 10^9^/L or granulocytes < 1 × 10^9^/L or platelets < 50 × 10^9^/L. Severe GI (grade ≥ 3) consisted in painful stomatitis or diarrhea (with more than 7 stools/day, or bloody stools, or incontinence, or severe cramps), and impaired enteral feeding.

**TABLE 1:** Demographic and clinical data of the study population.

	Italian cohort
Total patients	204
Age (*n* = 204)	
Median (IQR), years	4.85 (2.99–8.30)
Gender (*n* = 204)	
Male, *n*° (%)	113 (55.4)
Female, *n*° (%)	91 (44.6)
Ethnic group (*n* = 204)	
European ancestry, *n*° (%)	195 (95.6)
Others, *n*° (%)	9 (4.4)
Immunotyping (*n* = 202)	
LLA‐B, *n*° (%^a^)	182 (90.1)
LLA‐T, *n*° (%^a^)	20 (9.9)
Risk class (*n* = 204)^b^	
Standard, *n*° (%)	74 (36.3)
Medium, *n*° (%)	130 (63.7)
High, *n*° (%)	0 (0.0)
Toxicities during consolidation (Grades 3–5^c^, *n* = 88)	
HEM, *n*° (%^a^)	43 (48.9)
GI, *n*° (%^a^)	14 (15.9)
Infections, *n*° (%^a^)	4 (4.5)
Pancreatitis, *n*° (%^a^)	3 (3.4)
Renal toxicity, *n*° (%^a^)	2 (2.3)
Neurological toxicity, *n*° (%^a^)	1 (1.1)
Cardiotoxicity, *n*° (%^a^)	0 (0.0)
Outcome (*n* = 200)	
Complete remission (%^a^)	173 (86.5)
Relapse, *n*° (%^a^)	27 (13.5)
Median (IQR) in years to relapse	2.6 (1.7–3.3)
Follow‐up	
Median (IQR) in years since diagnosis, *N*	5.2 (4.6–6.4), 199

Abbreviations: GI, gastrointestinal toxicity; HEM, hematological toxicity; IQR, interquartile range; LLA‐B, LLA‐immunophenotype B; LLA‐T, LLA‐immunophenotype T; *n*°, numbers; Na, not available.

^a^ Percentage compared to available data.

^b^ Risk range at the end of the induction phase.

^c^ Severe HEM toxicities (grade ≥ 3) consisted in bone marrow suppression, with episodes of hemoglobin < 8 g/dL or white blood cells (WBC) < 2 × 10^9^/L or granulocytes < 1 × 10^9^/L or platelets < 50 × 10^9^/L. Severe GI (grade ≥ 3) consisted in painful stomatitis or diarrhea (with more than 7 stools/day, or bloody stools, or incontinence or severe cramps), and impaired oral feeding.

### 2.5 Statistical Analysis

Statistical analyses were performed using the software R version 3.5.2. MTX plasma levels and clearance were calculated for each cycle and considered as average measurement per patient, when not otherwise written. Intra‐patient variability of PK parameters was assessed as coefficient of variation (CV). PK parameters were analyzed both as continuous variables and as quartiles (i.e., Q_1_–Q_4_; Q_1_ representing the lower quartile and Q_4_ the upper one); MTX systemic exposure was considered “high” either when MTX plasma levels correspond to upper values (continuous data)/Q_4_ (categorical data) or when the MTX clearances correspond to lower values (continuous data)/Q_1_ (categorical data), this latter case corresponding to a delayed drug elimination. Normality of continuous data was examined using the Shapiro–Wilk test. The association between PK parameters (dependent variable) and pharmacogenomic and demographic variables (independent variables) was examined using linear models of the Gaussian family and linear mixed effect models; pharmacogenetic contribution were evaluated according to additive, dominant and recessive models of the minor alleles on the phenotype of interest. Age group were defined according to a cutoff value, arbitrarily set at 10 years, to account for different hormonal/developmental stage of patients (“children” vs. “adolescents”) and for survival disadvantage and increased occurrence of drug‐related adverse effects observed in older pediatric ALL patients compared to younger ones [19, 20].

The follow‐up time was calculated from diagnosis to the date of last available contact or the occurrence of an event (relapse, death, or second neoplasm), whichever came first. The Cox proportion hazard model was adopted for evaluating the impact of genotypes and MTX systemic exposure/clearance/intra‐patient variability on relapse.

Treatment‐related HEM and GI toxicities in consolidation were considered as dependent variables. Other drug‐related adverse effects incidence lower than 5% were not considered in the analysis. Adverse effects were dichotomized as severe (grades 3–5) versus non‐severe (grades 1–2 or absent), without discriminating among specific adverse episodes. Kruskal–Wallis test was used to investigate the influence of pharmacokinetic parameters on HEM and GI toxicities, considered separately. Associations between categorical variables were explored via Fisher's exact test (i.e., SNP genotypes on HEM and GI).

## 3 Results

### 3.1 Patient Characteristics and Pharmacological Analysis: SNP Genotyping and MTX Pharmacokinetics

Patient characteristics are shown in Table 1. Genotype distributions of candidate SNPs are shown in Table S2.

Pharmacokinetic parameters of 798 HD‐MTX courses in 200 patients are reported in Table S3. Twelve patients could not tolerate HD‐MTX infusions at the planned dose of 5 g/m^2^: Six patients reduced HD‐MTX from 2nd infusion, other four patients from 3rd on, and two patients received lower dosages at 4th infusion (28 courses in total). By protocol, the expected end‐of‐infusion concentration at 24 h should be lower than 150 μM; five patients exceeded this concentration in five courses (0.6% of total infusions). Median plasma MTX level at 24 h was 41.09 μM (interquartile range [IQR]: 31.68–53.70 μM), with similar intercourse values (Table S3, Figure S1A); intra‐patient variability in end‐of‐infusion concentrations was 31.3% (22.71%–45.72%). Post‐infusion MTX levels at 42 and 48 h were 0.54 (0.39–0.88) μM (798 samples in 200 patients) and 0.39 (0.28–0.63) μM (530 samples in 169 patients), respectively. Intercourse analysis showed decreased drug concentrations at both time points in late HD‐MTX cycles (*p* < 10^−4^ and *p* = 0.002, respectively, Kruskal–Wallis test, Table S3 and Figure S1B,C). About 15% of MTX plasma levels were above the expected 1 μM threshold at 42 h and about 29% above the expected 0.5 μM at 48 h; patients with exceeding values received according to the therapeutic protocol higher leucovorin rescue. Calculated MTX clearance in 200 patients was 159.80 (123.76–192.04) mL/min/m^2^, with similar median values among HD‐MTX cycles (Figure S1D). Intra‐patient variability in MTX clearance was 24.20% (16.03%–32.25%).

### 3.2 MTX Plasma Concentrations Are Higher in Older Patients and ABCC4 rs7317112 Wild‐Type Carriers

**FIGURE 2:**
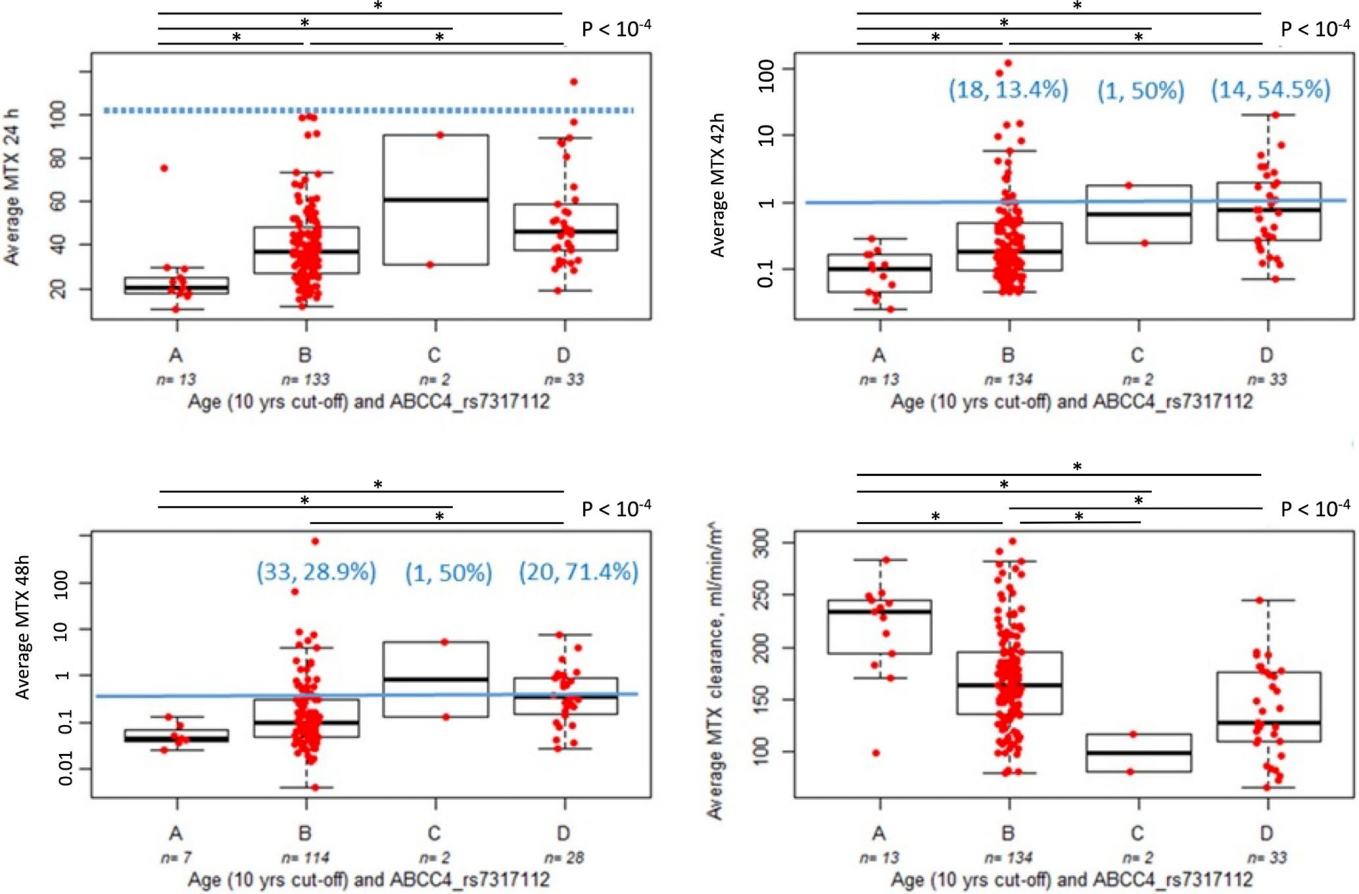
Methotrexate systemic exposure according to age and *ABCC4* SNP rs7317112. Patients were grouped according to age (dichotomized as “children” vs. “teens” by an arbitrary cutoff set at 10 years) and *ABCC4* SNP rs7317112 genotypes (dichotomized as mutated GG vs. non mutated), resulting in group A (GG children), group B (non‐GG children), group C (GG teens), and group D (non‐GG teens). Blue lines indicate the expected thresholds level of MTX at different time points; blue numbers in parentheses above the box plots indicated the number of patients (and percentage) above thresholds who received intensified leucovorin rescue. *p*‐value calculated according to Kruskal–Wallis test; + significance according to the Dunn test for multiple comparison.

MTX overall systemic exposure was affected by age, with older patients showing higher drug plasma levels at the end of infusion and delayed elimination compared to younger children (Table 2, Figure S2). Gender did not affect MTX pharmacokinetic parameters. Among candidate SNPs, *ABCC4* rs7317112 variant significantly reduced HD‐MTX exposure in univariate analysis (Table 2, Figure S3). MTX plasma concentrations were lower in homozygous variant GG patients compared to those carrying at least one A wild‐type allele, both at 24 h (Figure S3A) and at 42 h (Figure S3B). *ABCC4* rs7317112 variant resulted in an accelerated elimination of the drug (Figure S3D). Multivariate analysis confirmed the independent contribution of age and *ABCC4* SNP rs7317112 on pharmacokinetic parameters (Table 2). When patients were grouped according to age (cutoff arbitrarily set at 10 years) and *ABCC4* SNP rs7317112 (GG/non‐GG genotypes), MTX plasma levels at 24, 42, and 48 h were lower for young rs7317112 GG patients (group A) compared to rs7317112 non‐GG teens (group D), with a clearly higher clearance of the drug (Figure 2).

**TABLE 2:** Multivariate analysis on average end‐of‐infusion methotrexate plasma levels and clearance in the study population.

MTX plasma levels (24 h)	Univariate					Multivariate					*p* ^a^
	Coefficient	SE	95% CI		*p*	Coefficient	SE	95% CI		*p*	
Age (each year)	0.01	0.002	0.01	0.01	4.58 × 10^−6^	0.01	0.002	0.01	0.02	3.11 × 10^−7^	3.66 × 10^−12^
Gender (female vs. male)	0.03	0.02	−0.003	0.07	0.07						
*SLCO1B1* rs2306283 (non GG vs. GG)	0.06	0.02	0.007	0.107	0.03	0.04	0.02	−0.01	0.09	0.10	
*SLCO1B1* rs4149056 (non CC vs. CC)	−0.09	0.05	−0.19	0.016	0.09						
*ABCB1* rs1045642 (non GG vs. GG)	−0.03	0.02	−0.07	0.02	0.24						
*ABCC2* rs717620 (TT vs. non TT)	−0.03	0.05	−0.12	0.07	0.58						
*ABCC3* rs9895420 (non AA vs. AA)	0.10	0.08	−0.05	0.25	0.19						
*ABCC4* rs7317112 (non GG vs. GG)	0.17	0.03	0.10	0.24	4.61 × 10^**−7**^	0.16	0.03	0.10	0.23	1.04 × 10^**−6**^	

Clearance	Univariate					Multivariate					*p*
	Coefficient	SE	95% CI		*p*	Coefficient	SE	95% CI		*p*	
Age (each year)	−3.10	0.55	−4.19	−2.02	2.98 × 10^**−8**^	−3.32	0.58	−4.46	−2.02	1.57 × 10^**−8**^	1.64 × 10^**−12**^
Gender (female vs. male)	−6.48	4.62	−15.54	2.58	0.16						
*SLCO1B1* rs2306283 (non GG vs. GG)	−16.21	6.39	−28.73	−3.68	1.14 × 10^**−2**^	−13.12	6.51	−28.73	−3.68	4.43 × 10^**−2**^	
*SLCO1B1* rs4149056 (non CC vs. CC)	31.78	13.53	5.26	58.30	1.90 × 10^**−2**^	17.16	14.87	5.26	58.30	0.25	
*ABCB1* rs1045642 (non GG vs. GG)	2.78	5.59	−8.17	13.74	0.62						
*ABCC2* rs717620 (TT vs. non TT)	5.47	11.828	−17.71	28.66	0.64						
*ABCC3* rs9895420 (non AA vs. AA)	−20.58	19.11	−58.04	16.88	0.28						
*ABCC4* rs7317112 (non GG vs. GG)	−38.74	8.63	−55.67	−21.82	8.39 × 10^**−6**^	−36.25	8.46	−52.83	−21.82	2.06 × 10^**−5**^	

*Note:* Genetic contribution is presented according to a recessive model of minor allele (mutated vs. non mutated genotypes).

Abbreviations: CI, 95% confidence interval; SE, Standard error.

^a^ Likelihood ratio test. The coefficient of the interaction term between age and *ABCC4* rs7317112 was 6.3 (*p* = 6.39 × 10^−3^) for clearance.

Both SNPs in *SLCO1B1* were associated with pharmacokinetic parameters in univariate analysis, with opposite contribution of variant homozygous genotype on MTX plasma levels and clearance; however, only rs2306283 retained a borderline association with clearance after multivariate analysis.

### 3.3 Higher MTX Systemic Exposure and ABCC2 rs717620 Variant Allele Increased Risk of Severe Hematological Toxicities in Consolidation

The incidence of HEM (*n* = 43 (48.9%)) and GI (*n* = 14 (15.9%)) toxicity was not affected by age or gender (Table 3). The T‐variant allele of the *ABCC2* SNP rs717620 was a risk allele for both HEM and GI severe toxicities (wild‐type CC vs. CT + TT carriers: HEM: 37.7% vs. 74.1%, Fisher test: *p* = 4.1 × 10^−3^; GI: 7.5% vs. 29.6%, *p* = 1.7 × 10^−2^). A higher incidence of HEM was observed for carriers of *SLCO1B1* rs2306283 GG variant genotype compared to those carrying at least one wild‐type A allele (55.9% vs. 18.2%, respectively, *p* = 0.03).

**TABLE 3:** Pharmacogenetics contribution on patient's toxicities.

	Tot	HEM				GI			
		Yes (%)	OR	CI	*p*	Yes (%)	OR	CI	*p*
Age									
≥ 10 years	66	32 (48.5)	1.06	0.34–3.27	1	10 (15.1)	1.39	0.28–5.71	0.73
< 10 years	20	10 (50.0)				4 (20.0)			
Gender									
M	57	24 (42.1)	2.16	0.82–5.90	0.11	10 (17.5)	0.70	0.15–2.73	0.76
F	31	19 (61.3)				4 (12.9)			
*SLCO1B1* rs2306283									
Non‐GG	11	2 (18.2)	5.58	1.04–56.9	0.03	1 (9.1)	1.71	0.20–82.2	1
GG	68	38 (55.9)				10 (14.7)			
*SLCO1B1* rs4149056									
Non‐CC	3	2 (66.6)	0.48	0.01–9.55	0.62	1 (33.3)	0.34	0.02–21.8	0.39
CC	73	37 (50.1)				11 (14.5)			
*ABCB1* rs1045642									
Non‐GG	59	27 (45.8)	0.52	0.16–1.60	0.31	9 (15.2)	1.08	0.23–6.88	1
GG	21	13 (61.9)				3 (14.3)			
*ABCC2* rs717620									
Non‐CC	27	20 (74.1)	4.62	1.54–15.39	4.11 × 10^−3^	8 (29.6)	5.04	1.18–25.6	1.73 × 10^−2^
CC	53	20 (37.7)				4 (7.5)			
*ABCC3* rs9895420									
Non‐AA	71	36 (50.7)	NA	NA	NA	10 (14.1)	NA	NA	NA
AA	0	0 (0.0)				0			
*ABCC4* rs7317112									
Non‐GG	71	36 (50.7)	0.34	0.01–4.56	0.62	11 (15.5)	0.56	0.04–31.46	0.51
GG	4	3 (75.0)				1 (25.0)			
CL mean									
Q1	36	18	1.04	0.40–2.69	1	4	0.49	0.10–1.91	0.38
Q2–Q4	49	24				10			

Abbreviations: GI, Gastrointestinal toxicities; HEM, Hematological toxicities.

Increased systemic exposure to MTX, indicated by mean clearance values per patient in the lower quartile (Q1) was not associated with HEM or GI occurrence during consolidation (Table 3). However, when each infusion was analyzed separately, patients who developed HEM showed higher end‐of‐infusion plasma levels (Figure S4A, *p* = 0.013) and lower clearance (Figure S4B, *p* = 0.010) after the first course compared to those who did not. A tendency toward higher plasma concentration (Figure S4C, *p* = 0.052) and a significant delay in drug elimination (Figure S4D, *p* = 0.02) was observed also after the second course. No association was found with pharmacokinetic parameters of subsequent cycles.

After multiple corrections, only the association between *ABCC2* SNP rs717620 and HEM remained significant (*p* < 0.005).

### 3.4 SLCO1B1 SNP rs4149056 Decreased the Risk of Relapse

The impact of demographic variants on patients' outcomes was analyzed, showing a significant association of age, when patients were categorized as younger or older than 10 years. In particular, older children showed a 2.39 hazard risk (HR) or relapse (confidence interval [CI]:1.07–5.32, *p* = 0.03, Table 4). The impact of genetic variants on patients' outcomes was first analyzed by using an additive genetic model, suggesting a contribution of *SLCO1B1* SNP rs4149056 on relapse risk (Table 4). When a dominant model for the minor allele was applied, patients with at least one rs4149056 variant C‐allele showed a reduced risk of relapse compared to wild‐type TT carriers (hazard risk [HR]: 0.29, 95% CI: 0.09–0.98, *p* = 0.047). Other genetic variables were not significantly associated with relapse risk. To evaluate the possible impact of MTX exposure on patients' outcomes, mean MTX plasma levels at the end of infusion and clearance were considered as quartile (Q1–Q4). MTX systemic exposure was considered “high” when end‐of‐infusion MTX fell in the upper quartile (mean MTX (24 h)‐Q4) and clearance in the lower one (mean CL‐Q1). A delayed clearance (mean CL‐Q1) doubled the risk of relapse (HR: 2.3, CI: 1.06–5.12, *p* = 0.035, Table 4). The delayed clearance did not affect the number and/or the doses of subsequent courses of HD‐MTX administered (data not shown); however, 26 out of 39 patients with delayed clearance (66.6% mean CL, Q1) and 53 out of 135 patients in the other group (39.2% mean CL, Q2–Q4) require to continue leucovorin rescue at 48 h in at least one course because of MTX plasma levels exceeding 0.5 μM: the difference was significant (OR: 4.25, 95%CI: 1.98–9.59, Fisher test, *p* = 5.0 × 10^−5^). Low systemic exposure (meant as mean MTX (24 h)‐Q1 and mean CL‐Q4) was not associated with relapse risk.

**TABLE 4:** Pharmacological parameters and patient's relapse risk.

	*N* patients (relapse)	Univariate			Multivariate		
		HR (95% CI)	*p*	*p* ^a^	HR (95% CI)	*p*	*p* ^a^
Age							
< 10 years	156 (18)	1.0		0.04	1.0	0.08	0.01
≥ 10 years	36 (9)	2.39 (1.07–5.32)	0.03		2.14 (0.90–5.05)		
Gender							
M	106 (17)	1.0		0.3			
F	86 (10)	1.47 (0.67–3.22)	0.33				
*SLCO1B1* rs2306283							
AA	59 (5)	1.0		0.2			
AG	92 (17)	2.28 (0.83–6.21)	0.11				
GG	29 (3)	1.16 (0.28–4.88)	0.83				
*SLCO1B1* rs4149056							
TT	128 (22)	1.0		0.04	1		
TC	49 (2)	0.22 (0.05–0.93)	0.04		—		
CC	6 (1)	0.88 (0.12–6.62)	0.90		0.27 (0.08–0.90)^b^	0.037	
*ABCB1* rs1045642							
GG	40 (6)	1.0		0.9			
GA	94 (12)	0.82 (0.31–2.19)	0.98				
AA	49 (8)	1.01 (0.35–2.93)	0.69				
*ABCC2* rs717620							
CC	123 (19)	1.0		0.8			
CT	53 (6)	0.73 (0.29–1.85)	0.51				
TT	6 (1)	0.95 (0.13–7.16)	0.96				
*ABCC3* rs9895420							
TT	122 (19)	1.0		0.4			
TA	48 (5)	0.64 (0.24–1.71)	0.38				
AA	3 (1)	2.80 (0.37–21.02)	0.32				
*ABCC4* rs7317112							
AA	94 (13)	1.0		0.7			
AG	66 (8)	0.83 (0.34–2.01)	0.68				
GG	15 (3)	1.42 (0.40–5.00)	0.58				
MTX_24h_mean (quartile)							
Q1–Q3	142 (15)	1.0		0.10			
Q4	49 (11)	1.96 (0.88–4.37)	0.10				
CL_mean (quartile)							
Q2–Q4	142 (14)	1.0		0.04	1		
Q1	49 (12)	2.33 (1.06–5.12)	0.04		2.18 (0.93–5.07)	0.07	

^a^ Likelihood ratio test.

^b^ As dominant model of variant allele.

**FIGURE 3:**
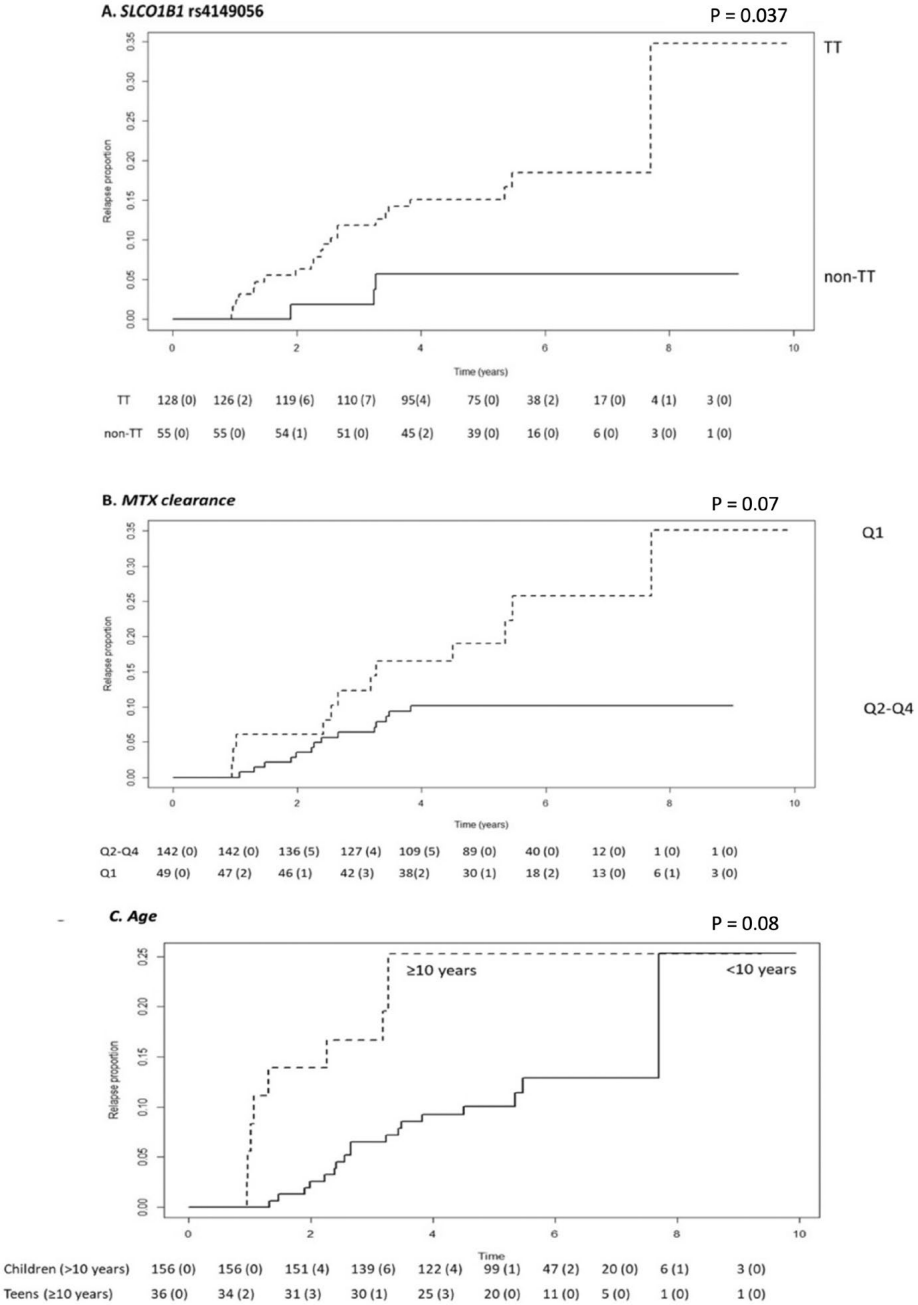
Impact of pharmacological parameters on relapse risk. (A) *SLCO1B1* rs4149056; (B) MTX clearance expressed in quartile. Q1: 1st quartile (delayed clearance); Q2: 2nd quartile; Q3: 3rd quartile; Q4: 4th quartile; (C) age (cutoff 10 years). *p*‐value according to multivariate analysis.

The association between *SLCO1B1* SNP rs4149056 and relapse risk remained fully significant after multivariate analysis (protective non‐TT genotype: HR: 0.27, CI: 0.08–0.90, *p* = 0.037), whereas older age and lower clearance retained borderline significance as adverse factors (HR: 2.14 (0.90–5.05), *p* = 0.08 and 2.18 (0.93–5.07), *p* = 0.07, respectively, Table 4, Figure 3).

## 4 Discussion

In ALL therapy, the importance of consolidating the early remission of the disease by removing undetectable residual tumoral cells is well recognized; optimization of drug intensities in this therapeutic phase is therefore mandatory to achieve a sustained event‐free survival. The use of oral MP combined to infusions of HD‐MTX is common in the consolidation phase, although substantial differences in MP/HD‐MTX dosages and timing of administrations are present across ALL protocols of different cooperative groups. Pharmacogenetic and pharmacokinetic evidences generated to improve drug therapy are thus protocol‐based, and are difficult to translate among ALL trials without an appropriate validation [21].

Genetic variability in HD‐MTX pharmacokinetics and toxicities has been extensively investigated, but without conclusive results [22, 23]. *MTHFR* SNPs rs1801133 and rs1801131, involved in MTX pharmacodynamics, are the best characterized variants [23, 24]; however, these SNPs reach only a level of evidence 3 for drug–gene association in Pharmacogenomics Knowledge Base (PharmGKB) database, and are excluded from the two highest rankings of evidence selected by Pharmacogenomic consortia (the Dutch Pharmacogenetics Working Group [DPWG] and the Clinical Pharmacogenetics Implementation Consortium [CPIC]) to develop pharmacogenetic‐based guideline [25]. This is likely due to several reasons including, among others, the low‐penetrance of variants considered, the variety and complexity of metabolic pathways with which MTX interferes, and the complex clinical traits taken into account. In contrast, pharmacogenetic‐based guidelines for MP are currently available since the role of *TPMT* and *NUDT15* gene variants on MP intolerances is well established [25], and additional contributions of variants in *ITPA* and *PACSIN2* genes are under investigation [26, 27]. Pharmacokinetic variability in HD‐MTX might also affect both therapy efficacy and toxicity. Post‐infusions high interindividual variability in MTX levels have been reported in several studies, and confirmed in our study cohort. Because an adequate clearance is fundamental to maintain MTX plasma level in the therapeutic window, preventing drug adverse reactions or worst outcome, this study evaluated the impact of different pharmacogenetic variants in influx–efflux drug transporters on HD‐MTX PK and clinical outcome in ALL pediatric patients for the first time in the specific context of the AIEOP‐BFM ALL 2009 protocol.

AIEOP‐BFM ALL 2009 protocol was in force in Italy between October 01, 2010 and February 28, 2017 enrolling 2098 patients (1576 [75%] not at high risk). With 204 patients, our study population represent a small subset cohort of AIEOP‐BFM ALL 2009 treated patients; however, features such as age, gender, immunophenotype, and outcome are in accordance with those previously reported [28, 29], suggesting that our results might be representative for the whole cohort.

Interestingly, a contribution of age on drug systemic exposure was observed, as indicated by a delayed elimination of MTX and the need of higher doses of leucovorin in teenagers. Previous studies have already shown that systemic exposure to MTX is higher in adolescents than in children [15]. In the study of Zobeck et al., older age conferred an increase in the odds of using carboxypeptidase G2 (CPDG2; glucarpidase), a bacterial‐derived enzyme that can rapidly reduce serum levels of MTX by hydrolysis to inactive metabolite [30]. These results suggest a possible role of age‐related epigenetic factors in MTX disposition. Because DNA methylation changes in growing children, it may contribute to ontogeny of enzymes involved in drug biotransformation and of transporters involved in drug elimination [31, 32]. Different methylation levels in the MTX hydrolases GGH were associated with an altered expression of this gene and to a reduced enzyme activity, which affected the treatment response and was associated with MTX adverse reactions [33, 34]. Doses required in children are usually higher due to an increased metabolism or drug transport [35]. Besides age, the significant influence of the intronic *ABCC4* SNP rs7317112 on HD‐MTX PK was found, indicating that the presence of variant G allele in the renal efflux transporter gene prompted drug elimination. *ABCC4* SNP rs7317112 is located in an enhancer region and in a CpG site, which could carry changes in the methylation pattern, and thus in the MRP4 expression. The reduced drug clearance could alter the incidence of HD‐MTX side effects; however, in our study population, *ABCC4* SNP rs7317112 genotypes did not influence the occurrence of HEM or GI. A previous study on MTX‐induced mucositis showed a protective role of the G allele [36]. In the study of Zobeck et al., each additional G allele of rs7317112 in *ABCC4* was strongly associated with risk for requiring CPDG2, and thus likely with a reduced tubular excretion of drugs [30]. Grouping patients according to age (cutoff: 10 years) and *ABCC4* SNP rs7317112 could not help identifying *a priori* intolerant patients (those who will reduce the 5 g/m^2^ planned doses after the first HD‐MTX cycle, data not shown). However, it could predict patients who will not exceed the expected MTX plasma levels of 150 μM at 24 h, 1 μM at 42 h, and 0.5 μM at 48 h (i.e., rs7317112 GG children younger than 10 years) and those who will likely require an intensified leucovorin rescue (i.e., rs7317112 non‐GG teens). In perspective, an optimized HD‐MTX infusion scheme and supportive management tailored for this latter group could be proposed, although this group does not suffer of higher toxicities; however, an increased relapse rate was observed for teens and for patients with lower MTX clearance.

The results emerged in this study indicate *ABCC2* rs717620 (−24C>T) as a contributing factor in the HD‐MTX adverse reaction risk, in line with previous evidences [37, 38, 39, 40]. *ABCC2* encodes for multidrug resistance‐associated protein 2 (MRP2) that is expressed at the apical canalicular membrane of liver cells, kidney proximal tubule epithelial cells, enterocytes of the small and large intestine, and syncytiotrophoblasts of the placenta [41]. No effect on HD‐MTX clearance of the *ABCC2* rs717620 variant was found in our study population, suggesting that HEM toxicity was not related to excessive MTX systemic exposure, but rather to an accumulation of toxic levels of MTX in hematopoietic cells. Thiopurines are not substrate for MRP2 [42]; thus, the increased HEM toxicity observed for *ABCC2* rs717620 carriers is not likely related to accumulation of concomitant MP metabolites in hematopoietic stem cells. Conflicting results on the role of *ABCC2* rs717620 in MTX‐PK are reported in the literature, as reviewed by Taylor et al. [43], likely because there is no linear correlation between the −24T allele and the transporter functional expression. In *in vitro* report gene assays, the −24T allele was associated with both increased and decreased promoter function [44, 45]; *in vivo*, *ABCC2* rs717620 was reported in linkage disequilibrium (LD) with other *ABCC2* coding SNPs with uncertain impact on the efflux pump (i.e., rs2273697 (1249G>A, p.Val417Ile, *D*′ = 0.694) and rs3740066 (c.3972C>T, p.Ile1324=, *D*′ = 0.699)) [46]. Interestingly, *ABCC2* haplotype (−24T, 1249G, 3972T) was associated with imatinib resistance in chronic myeloid leukemia patients, suggesting a lower expression of MRP2 protein and reduced transport activity in haplotype carriers [47]. The association between *ABCC2* rs717620 and HD‐MTX adverse effects deserved further validation studies, and it is worth performing a deeper genetic characterization of *ABCC2* gene and its promoter in ALL patients.

Our results showed a protective role of the rs4149056 C variant allele toward relapse. To the best of our knowledge, this is the first study demonstrating an association between rs4149056 SNP in the *SLCO1B1* gene and relapse risk in a Caucasian population of pediatric ALL patients treated according to the AIEOP‐BFM ALL guidelines. GWAS studies conducted on ALL pediatric patients identified SNPs in the *SLCO1B1* gene, encoding for the OAT1B1 MTX‐influx transporter and localized in the sinusoidal membrane of hepatocytes, as the major genetic marker of HD‐MTX pharmacokinetic variability [15, 48]. Contribution of rare damaging nonsynonymous variants in *SLCO1B1* was also demonstrated [15]. Moreover, a mouse model using a knockout (KO) of the murine ortholog, *Slco1b2*, confirmed a compromised transport of MTX into hepatocytes and a decreased drug clearance [49]. The association between rs4149056 and PK parameters, although replicated in many ALL patients' cohorts [37, 50] did not emerge in our study population of mainly Caucasian patients likely because of different genetic backgrounds, lifestyles, or treatment protocol. Previous studies on Saint Jude Children's Research Hospital trials associated the rs4149056 minor C allele to a decreased risk of MTX‐induced gastrointestinal complications in ALL patients, suggesting the contribution of the SNP as limiting for the biliary excretion of the drug into the intestine [48]. Again, controversial results on the impact of this genetic variant on other MTX‐related toxicities are reported in the literature [51] and were not evident in our study cohort. Discrepancy among results is probably due to difference in ethnicity of study populations, protocol treatments, and cohort size considered. We cannot rule out the hypothesis that the genetic contribution of rs4149056 on relapse is affecting the prolonged systemic exposure of MTX during maintenance rather than during consolidation. We reasoned that variability in the minor biliary excretion route of MTX (accounting for around 10%–30% of MTX clearance) becomes somehow more important when hepatic metabolic enzymes (i.e., aldehyde oxidase generating inactive 7‐hydroxymethotrexate) and hepatic and renal transporters are not saturated by the drug, as it is likely to happen after low doses of MTX.

This study is an initial explorative retrospective study that focus on a limited number of SNPs in the MTX transport pathway. In particular, the study did not include the characterization of MTHFR SNPs rs1801133, which could be a limitation, especially when interpreting the association with outcome data. Future research could involve a more comprehensive characterization of genetic contributions to HD‐MTX pharmacokinetics using a GWAS approach. Moreover, in future studies, the current PK model could be further refined by incorporating important variables such as patients' kidney and liver function, age, and certain genetic variants. In conclusion, these data highlighted the importance of variants in drug transporters genes on HD‐MTX disposition in the AIEOP‐BFM ALL 2009 protocol consolidation phase, and their putative role as predictive markers of outcome. Age and *ABCC4* SNP rs7317112 affect HD‐MTX PK parameters, *ABCC2* SNP rs717620 could play a role as predictive marker of HD‐MTX‐induced HEM in consolidation and *SLCO1B1* SNP rs4149056 as predictive marker of relapse, if validated in additional AIEOP‐BFM cohorts. Further prospective studies will be required for the clinical application in particular of *ABCC2* SNP rs717620.

## Author Contributions

R.F., S.B., and G.Z. wrote the manuscript. R.F., G.S., G.D., and M.R. designed the research. R.F., S.B., G.Z., V.K., M.R., A.C., L.R.B., E.B., F.L., L.V., N.B., F.F., D.S., and M.G.V. performed the research. R.F., S.B., G.Z., and G.S. analyzed the data.

## Conflicts of Interest

The authors declare no conflicts of interest.

## Supporting information


Data S1.

## Data Availability

The original contributions presented in this manuscript are included in the article. Further inquiries can be directed to the corresponding author.
