# Supplementary material for: The Role of Candidate Polymorphisms in Drug Transporter Genes on High‐Dose Methotrexate in the Consolidation Phase of the AIEOP‐BFM ALL 2009 Protocol
Source: Clin Transl Sci. 2025 Jan 31;18(2):e70136. doi: 10.1111/cts.70136 (PMC11786019; doi:10.1111/cts.70136)
Supplement: Supplementary file 1 — Data S1. [file CTS-18-e70136-s001.docx]

**SUPPLEMENTARY MATERIAL**

| ***SLCO1B1*** rs2306283  (388 A>G) | **Var G:** controversial impact on OATP1B1  **Var G: ↑ MTX CL** significant only in ALL patients carrying the lower-function rs4149056 T521C allele  **Var G: ↑ [MTX]48h ↑ tox in delay excretion group**  **Allele A: ↓ MTX CL**  **Var G: does not affect [MTX], MTX response and toxicity**  **Allele A: ↑ MTX CL** | ^1^  ^1^  ^2^  ^3^  ^4^  ^5^  ^5^ |
| --- | --- | --- |
| ***SLCO1B1*** rs4149056  (521 T>C) | **Var C: markedly decreases OATP1B1 function**  **var**  **C**: **↓ MTX CL** (for each additional C allele) **and ↑ GI toxicity in ALL**  **var**  **C**: **↓ MTX CL and ↑ tox**  **var**  **C**: **↑ GI toxicity AND ↓ hepatotoxicity in JIA patients**  **varC: ↓ EFS =tox**  rs4149081 and rs11045879 SLCO1B1 and MTX plasma concentration  **no effect on MTX PK**  **var**  **C**: does not affect **MTX response**  **var**  **C**: **↓ MTX CL**  **Allele T: ↓ [MTX]**  **var**  **C**: **↑ MTX toxicity (thrombocytopenia, mucositis)**  **Allele T: does not affect MTX toxicity**  **var**  **C**: **↓ MTX response**  **var**  **C**: **↓ [MTX]** | ^6^  ^6^  ^7^  ^8^  ^4^  ^9^  ^10^  ^11^  ^5^  ^12^  ^13^  ^4^  ^4^  ^14^ |
| ***ABCB1*** rs1045642 | Var: **↑ HEM in ALL patients**  Var: **no HEM, ↑ HEP in ALL patients**  **No effect on PK**  Var T: **↓ MTX CL and ↑ MTX toxicity** Var T: **↓ MTX CL and ↑ MTX hematological toxicity and Event Free Survival rate**  Var T: **↓ MTX CL and ↑ MTX hematological toxicity**  Var T: **↑ MTX toxicity (mucositis)**  Var T: **↑ ALL development risk** | ^15^  ^16^  ^9^  ^17^  ^18^  ^19^  ^20^  ^21^ |
| ***ABCC2*** rs717620  (-24 C>T) | Var T: increased promoter function  Var T: **↑ MTX AUC (↓CL)**  Var T: **↑ [MTX]48h, ↑ HEM , ↑ GI in ALL patients (Chinese)**  Var T: gender-specific impact on AUC  Var T: **↑ HEM** | ^22^  ^15^  ^23^  ^24^  ^10^ |
| ***ABCC3*** rs9895420  (-189T>A) | Var A: higher promoter activity  Hom Var; **↓ GI**  Var; **↓ HEM, ↑[MTX]24h, ↓EFS** | ^25^  ^25^  ^26^ |
| ***ABCC4*** rs7317112 | Wt A: **↑ mucositis**  **Var G: ↑odds of requiring CPDG2** | ^27^  ^28^ |

**Table S1:** influence of genetic variants of interest on MTX.

| **GENE** | **SNP** | **N°** | **Wt (n, %)** | **hz (n, %)** | **var (n, %)** | **HWE**  **P-value** |
| --- | --- | --- | --- | --- | --- | --- |
| ***SLCO1B1*** | rs2306283  c.388 A>G, p.N130D | 192 | 65 (33.85%) | 96 (50.0%) | 31(16.15%) | 0.736 |
| ***SLCO1B1*** | rs4149056  c.521 T>C, p.V174A | 195 | 139 (71.28%) | 50 (25.64%) | 6 (3.08%) | 0.728 |
| ***ABCB1*** | rs1045642  c.3435 C>T, p.I1145I | 195 | 52 (26.67%) | 95 (48.72%) | 48 (24.62%) | 0.805 |
| ***ABCC2*** | rs717620  -24 C>T | 194 | 130 (67.01%) | 56 (28.87%) | 8 (4.12%) | 0.668 |
| ***ABCC3*** | rs9895420  -189 T>A | 185 | 132 (71.35%) | 50 (27.03%) | 3 (1.62%) | 0.643 |
| ***ABCC4*** | rs7317112  A>G, intron | 187 | 103 (55.08%) | 69 (36.9%) | 15 (8.02%) | 0.573 |

**Table S2**: **SNPs genotype distribution in the study populations.** SNP minor allele frequencies were as expected for Caucasians and genotype frequencies were in accordance with Hardy–Weinberg equilibrium (p>0.05). HWE: Hardy-Weinberg equilibrium; het: heterozygous; wt: wild type; Var: variant; N: total number of patients.

|  | total | 1.st course | 2.nd course | 3.rd course | 4.th course | p-value |
| --- | --- | --- | --- | --- | --- | --- |
| N | 798 | 200 | 200 | 200 | 198 | - |
| HD-MTX Dosage  5g/m^2^, N  4 g/m^2^, N  3.5 g/m^2^, N  3 g/m^2^, N  2.5 g/m^2^, N  2 g/m^2^, N | 770  2  8  1  4  13 | 200  0  0  0  0  0 | 194  0  1  0  2  3 | 190  0  4  1  0  5 | 186  2  3  0  2  5 | - |
| MTX plasma concentrations  (N=200)*  24h (median (IQR) in μM), N  ≥150 μM at 24h, n (%^+^) | 41.09 (31.68-53.70), 200 | 40.84 (27.60-57.00), 197  2 (1.0) | 40.00 (25.27-56.77), 199  0 (0.0) | 39.15 (26.79-55.35), 196  3 (1.5) | 36.31 (24.48-52.60), 194  0 (0.0) | Ns |
| 42h (median (IQR) in μM, N)  >1 μM at 42h, n (%^+^) | 0.54 (0.39-0.88), 200 | 0.53 (0.40-0.86), 197  36 (18.3) | 0.48 (0.33-0.86), 197  36 (18.3) | 0.44 (0.32-0.66), 199  24 (12.1) | 0.43 (0.32-0.72), 198  27 (13.6) | <10^-4^ |
| 48h (median (IQR) in μM, N)  >0.5 μM at 48h, n (%^+^) | 0.39 (0.28-0.63), 169 | 0.37 (0.29-0.70), 145  49 (33.8) | 0.37 (0.28-0.63), 126  44 (34.9) | 0.31 (0.24-0.47), 130  30 (23.1) | 0.32 (0.24-0.55), 129  38 (29.5) | 0.002  - |
| Clearance  mediana (IQR) in ml/min/m^2^, N | 159.80 (123.76-192.04), 200 | 150.16 (116.46-198.54), 200 | 147.17 (116.67-209.25), 200 | 148.97 (114.54-203.00), 200 | 158.01 (122.23-210.87), 198 | Ns |

**Table S3: MTX plasma concentrations and clearance.** Data referred to patients who received MTX infusion at 5g/m^2^ in first course; + percentage compared to available data IQR: interquartile range; N: total number of patients; NA: not assessed; Ns: non-significant. P-value according to the non-parametric kruskal wallis test.


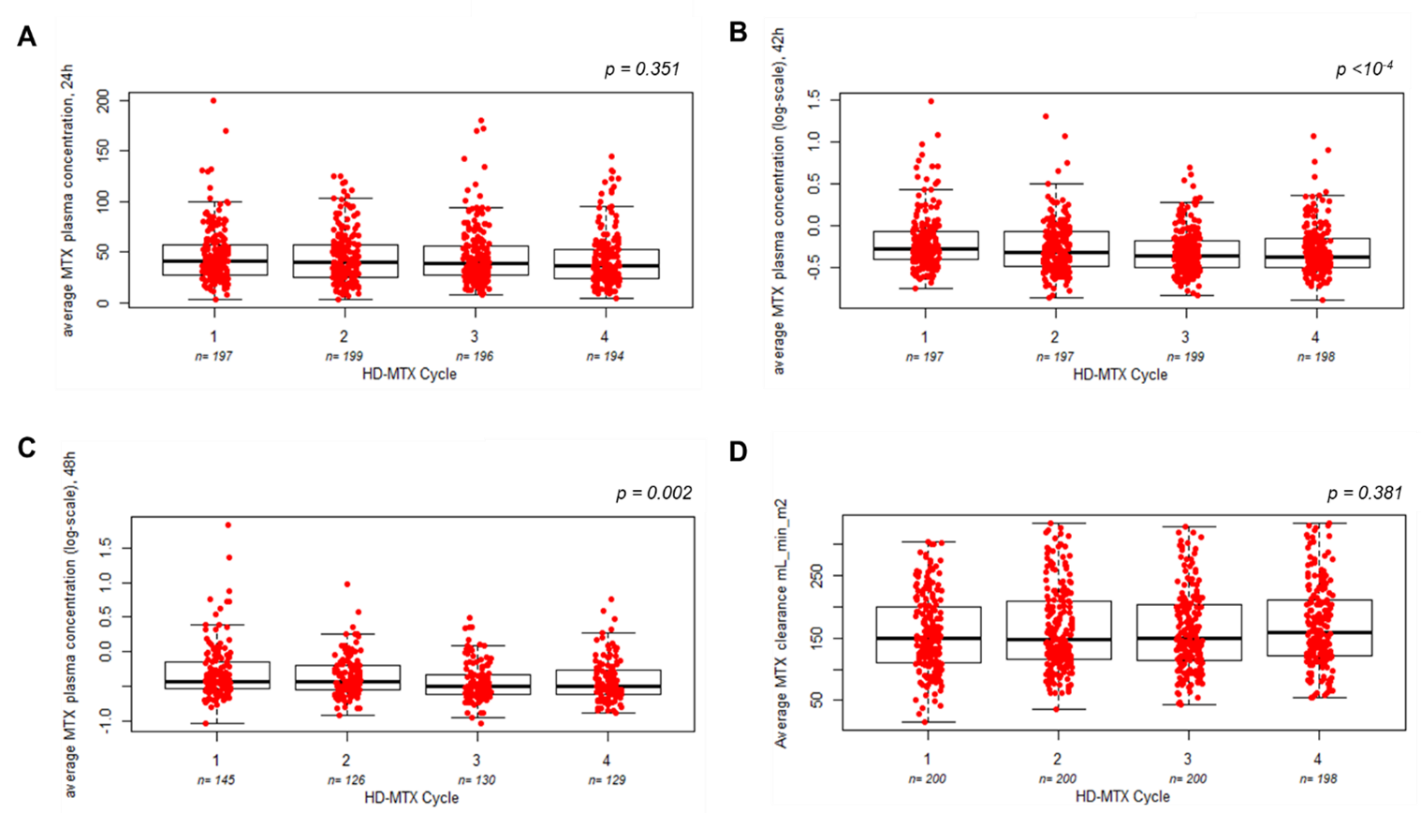


**Figure S1** **:** **MTX-HD plasma concentrations and clearance per cycle** in patients who received the 1.st HD-MTX course at planned dose of 5 g/m2. P-value calculated according to Kruskal Wallis test.


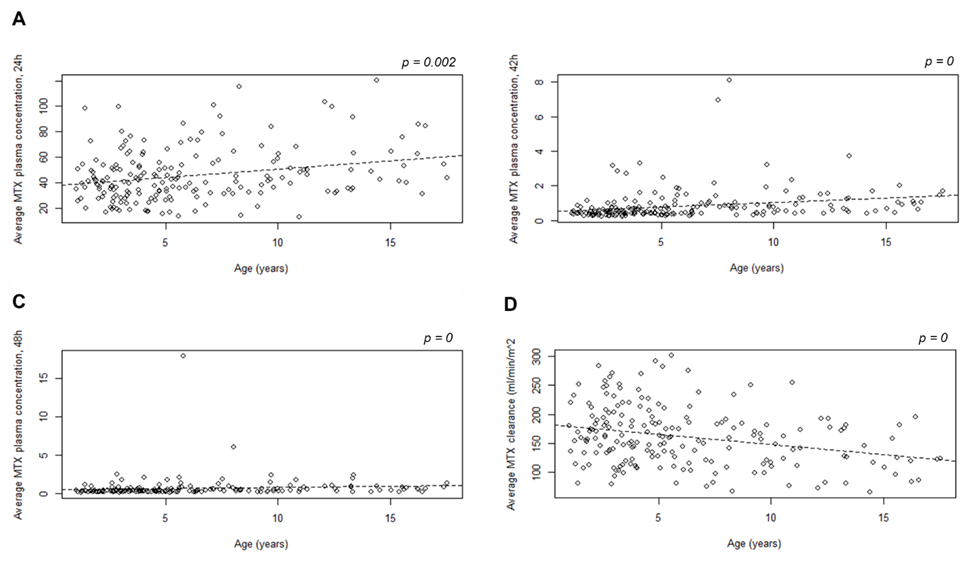


**Figure S2** **: Methotrexate overall exposure increased with age in the Italian cohort.** MTX plasma concentrations A) at 24 h; B) at 42h; C) at 48h and D) MTX clearance. P-value calculated according to Spearman test.


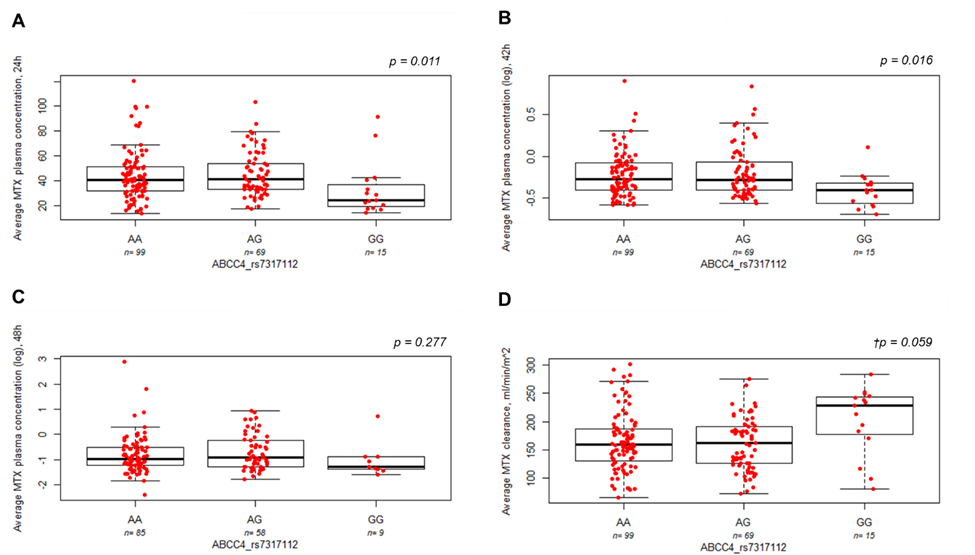


**Supplementary Figure S3** **: Methotrexate overall exposure is decreased in homozygous variant patients for ABCC4 SNP rs7317112 compared to wild type allele carriers**. MTX plasma concentrations A) at 24 h (GG genotype: median (interquartile range): 24.50 (19.37-37.06 μM; AG genotype: 41.35 (33.20- 53.84) μM; AA genotype: 40.55 (31.66-51.59) μM); B) at 42h (shown in log-scale, original values: GG: 0.39 (0.27-0.47) μM; AG: 0.52 (0.39-0.85) μM; AA: 0.53 (0.39-0.83) μM); C) at 48h (shown in log-scale) and D) MTX clearance (GG: 228.75 (176.78-243.78) ml/min/m^2^; AG: 162.60 (125.60-191.55) ml/min/m^2^; AA e: 159.75 (130.50-187.23) ml/min/m^2^). P-value calculated according to Kruskal Wallis test, †P-value calculated according to one-way ANOVA.


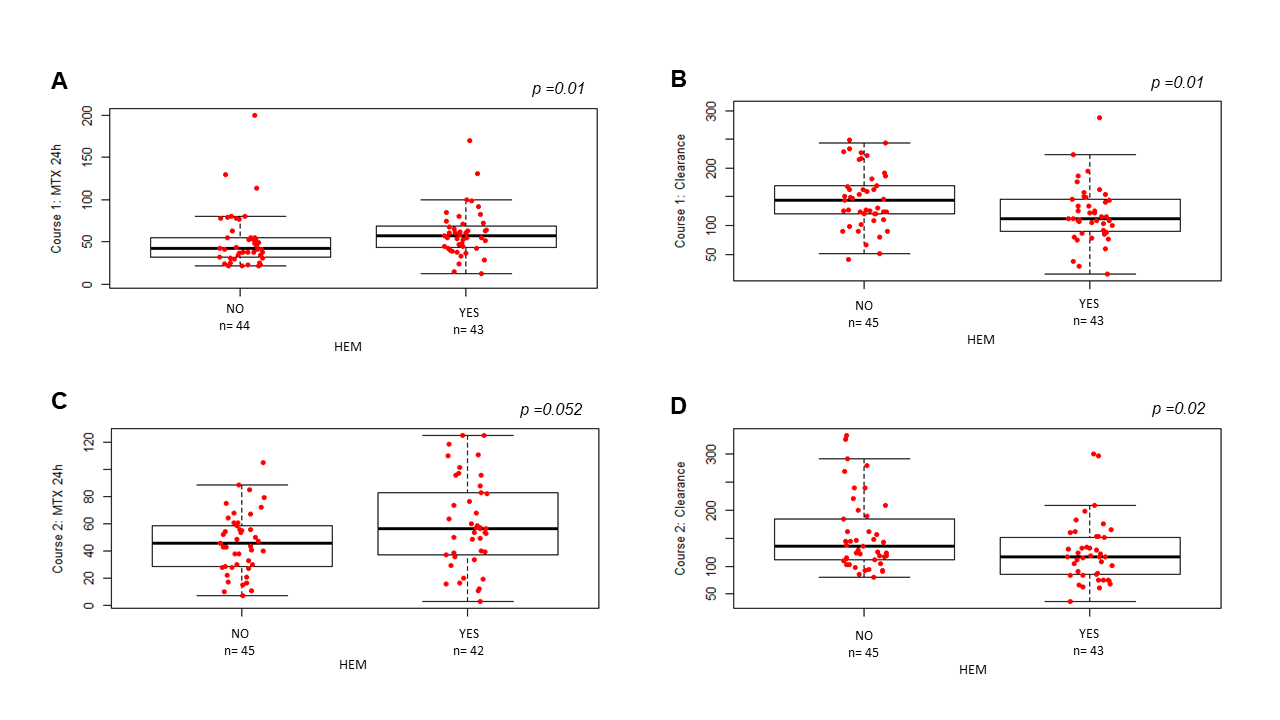


**Supplementary Figure S4** **: Methotrexate systemic exposure and hematological toxicities for (A, B) the first HD-MTX cycle and (C, D) the second HD-MTX cycle**  **PK parameters of each infusion were matched to HEM/GI toxicities regardless of their time of occurrence during consolidation.** P-value calculated according to Kruskal Wallis test.

**References supplementary**

1. Ramsey LB, Bruun GH, Yang W, et al. Rare versus common variants in pharmacogenetics: SLCO1B1 variation and methotrexate disposition. *Genome Res*. Jan 2012;22(1):1-8. doi:10.1101/gr.129668.111

2. Cheng Y, Chen MH, Zhuang Q, et al. Genetic factors involved in delayed methotrexate elimination in children with acute lymphoblastic leukemia. *Pediatr Blood Cancer*. May 2021;68(5):e28858. doi:10.1002/pbc.28858

3. Ramsey LB, Panetta JC, Smith C, et al. Genome-wide study of methotrexate clearance replicates SLCO1B1. *Blood*. Feb 07 2013;121(6):898-904. doi:10.1182/blood-2012-08-452839

4. Liu SG, Gao C, Zhang RD, et al. Polymorphisms in methotrexate transporters and their relationship to plasma methotrexate levels, toxicity of high-dose methotrexate, and outcome of pediatric acute lymphoblastic leukemia. *Oncotarget*. Jun 06 2017;8(23):37761-37772. doi:10.18632/oncotarget.17781

5. Radtke S, Zolk O, Renner B, et al. Germline genetic variations in methotrexate candidate genes are associated with pharmacokinetics, toxicity, and outcome in childhood acute lymphoblastic leukemia. *Blood*. Jun 27 2013;121(26):5145-53. doi:10.1182/blood-2013-01-480335

6. Treviño LR, Shimasaki N, Yang W, et al. Germline genetic variation in an organic anion transporter polypeptide associated with methotrexate pharmacokinetics and clinical effects. *J Clin Oncol*. Dec 10 2009;27(35):5972-8. doi:10.1200/JCO.2008.20.4156

7. Zhang HN, He XL, Wang C, et al. Impact of SLCO1B1 521T > C variant on leucovorin rescue and risk of relapse in childhood acute lymphoblastic leukemia treated with high-dose methotrexate. *Pediatr Blood Cancer*. Dec 2014;61(12):2203-7. doi:10.1002/pbc.25191

8. Roszkiewicz J, Michałek D, Ryk A, Swacha Z, Szmyd B, Smolewska E. variants as predictors of methotrexate-related toxicity in children with juvenile idiopathic arthritis. *Scand J Rheumatol*. May 2021;50(3):213-217. doi:10.1080/03009742.2020.1818821

9. Lopez-Lopez E, Martin-Guerrero I, Ballesteros J, et al. Polymorphisms of the SLCO1B1 gene predict methotrexate-related toxicity in childhood acute lymphoblastic leukemia. *Pediatr Blood Cancer*. Oct 2011;57(4):612-9. doi:10.1002/pbc.23074

10. Razali RH, Noorizhab MNF, Jamari H, et al. Association of A*BCC2*with levels and toxicity of methotrexate in Malaysian Childhood Acute Lymphoblastic Leukemia (ALL). *Pediatr Hematol Oncol*. Apr 2020;37(3):185-197. doi:10.1080/08880018.2019.1705949

11. Fukushima H, Fukushima T, Sakai A, et al. Polymorphisms of MTHFR Associated with Higher Relapse/Death Ratio and Delayed Weekly MTX Administration in Pediatric Lymphoid Malignancies. *Leuk Res Treatment*. 2013;2013:238528. doi:10.1155/2013/238528

12. Csordas K, Lautner-Csorba O, Semsei AF, et al. Associations of novel genetic variations in the folate-related and ARID5B genes with the pharmacokinetics and toxicity of high-dose methotrexate in paediatric acute lymphoblastic leukaemia. *Br J Haematol*. Aug 2014;166(3):410-20. doi:10.1111/bjh.12886

13. Yang FF, Xue TL, Gao C, et al. Effects of S*LCO1B1*on elimination and toxicities of high-dose methotrexate in pediatric acute lymphoblastic leukemia. *Pharmacogenomics*. Oct 2022;23(15):821-834. doi:10.2217/pgs-2022-0098

14. Eldem İ, Yavuz D, Cumaoğullari Ö, et al. SLCO1B1 Polymorphisms are Associated With Drug Intolerance in Childhood Leukemia Maintenance Therapy. *J Pediatr Hematol Oncol*. Jul 2018;40(5):e289-e294. doi:10.1097/MPH.0000000000001153

15. Zgheib NK, Akra-Ismail M, Aridi C, et al. Genetic polymorphisms in candidate genes predict increased toxicity with methotrexate therapy in Lebanese children with acute lymphoblastic leukemia. *Pharmacogenet Genomics*. Aug 2014;24(8):387-96. doi:10.1097/FPC.0000000000000069

16. Gregers J, Gréen H, Christensen IJ, et al. Polymorphisms in the ABCB1 gene and effect on outcome and toxicity in childhood acute lymphoblastic leukemia. *Pharmacogenomics J*. Aug 2015;15(4):372-9. doi:10.1038/tpj.2014.81

17. Ebid AIM, Hossam A, El Gammal MM, Soror S, Mangoud NOM, Mahmoud MA. High dose methotrexate in adult Egyptian patients with hematological malignancies: impact of ABCB1 3435C > T rs1045642 and MTHFR 677C > T rs1801133 polymorphisms on toxicities and delayed elimination. *J Chemother*. Oct 2022;34(6):381-390. doi:10.1080/1120009X.2021.2009723

18. Esmaili MA, Kazemi A, Faranoush M, et al. Polymorphisms within methotrexate pathway genes: Relationship between plasma methotrexate levels, toxicity experienced and outcome in pediatric acute lymphoblastic leukemia. *Iran J Basic Med Sci*. Jun 2020;23(6):800-809. doi:10.22038/ijbms.2020.41754.9858

19. Ramírez-Pacheco A, Moreno-Guerrero S, Alamillo I, Medina-Sanson A, Lopez B, Moreno-Galván M. Mexican Childhood Acute Lymphoblastic Leukemia: A Pilot Study of the MDR1 and MTHFR Gene Polymorphisms and Their Associations with Clinical Outcomes. *Genet Test Mol Biomarkers*. Oct 2016;20(10):597-602. doi:10.1089/gtmb.2015.0287

20. Gong Y, Luo L, Wang L, et al. Association of MTHFR and ABCB1 polymorphisms with MTX-induced mucositis in Chinese paediatric patients with acute lymphoblastic leukaemia, lymphoma or osteosarcoma-A retrospective cohort study. *J Clin Pharm Ther*. Dec 2021;46(6):1557-1563. doi:10.1111/jcpt.13505

21. Zaruma-Torres F, Lares-Asseff I, Lima A, et al. Genetic Polymorphisms Associated to Folate Transport as Predictors of Increased Risk for Acute Lymphoblastic Leukemia in Mexican Children. *Front Pharmacol*. 2016;7:238. doi:10.3389/fphar.2016.00238

22. Nguyen TD, Markova S, Liu W, et al. Functional characterization of ABCC2 promoter polymorphisms and allele-specific expression. *Pharmacogenomics J*. Oct 2013;13(5):396-402. doi:10.1038/tpj.2012.20

23. Liu Y, Yin Y, Sheng Q, et al. Association of ABCC2 -24C>T polymorphism with high-dose methotrexate plasma concentrations and toxicities in childhood acute lymphoblastic leukemia. *PLoS One*. 2014;9(1):e82681. doi:10.1371/journal.pone.0082681

24. Rau T, Erney B, Göres R, Eschenhagen T, Beck J, Langer T. High-dose methotrexate in pediatric acute lymphoblastic leukemia: impact of ABCC2 polymorphisms on plasma concentrations. *Clin Pharmacol Ther*. Nov 2006;80(5):468-76. doi:10.1016/j.clpt.2006.08.012

25. de Carvalho DC, Wanderley AV, Dos Santos AMR, et al. Pharmacogenomics and variations in the risk of toxicity during the consolidation/maintenance phases of the treatment of pediatric B-cell leukemia patients from an admixed population in the Brazilian Amazon. *Leuk Res*. Nov 2018;74:10-13. doi:10.1016/j.leukres.2018.09.003

26. Ansari M, Sauty G, Labuda M, et al. Polymorphism in multidrug resistance-associated protein gene 3 is associated with outcomes in childhood acute lymphoblastic leukemia. *Pharmacogenomics J*. Oct 2012;12(5):386-94. doi:10.1038/tpj.2011.17

27. den Hoed MA, Lopez-Lopez E, te Winkel ML, et al. Genetic and metabolic determinants of methotrexate-induced mucositis in pediatric acute lymphoblastic leukemia. *Pharmacogenomics J*. Jun 2015;15(3):248-54. doi:10.1038/tpj.2014.63

28. Zobeck MC, Bernhardt MB, Kamdar KY, Rabin KR, Lupo PJ, Scheurer ME. Novel risk factors for glucarpidase use in pediatric acute lymphoblastic leukemia: Hispanic ethnicity, age, and the ABCC4 gene. *Pediatr Blood Cancer*. Aug 2021;68(8):e29036. doi:10.1002/pbc.29036
